# Health Financing Consequences of Implementing Health Transformation Plan in Iran: Achievements and Challenges

**DOI:** 10.15171/ijhpm.2019.18

**Published:** 2019-04-13

**Authors:** Leila Doshmangir, Mohammad Bazyar, Behzad Najafi, Hassan Haghparast-Bidgoli

**Affiliations:** ^1^ Tabriz Health Services Management Research Center, Iranian Center of Excellence in Health Management, Tabriz University of Medical Sciences, Tabriz, Iran.; ^2^ Social Determinants of Health Research Center, Health Management and Safety Promotion Research Institute, Tabriz University of Medical Sciences, Tabriz, Iran.; ^3^ Department of Health Services Management, School of Management and Medical Informatics, Tabriz University of Medical Sciences, Tabriz, Iran.; ^4^ Department of Public Health, Faculty of Health, Ilam University of Medical Sciences, Ilam, Iran.; ^5^ Tabriz Health Services Management Research Center, Tabriz University of Medical Sciences, Tabriz, Iran.; ^6^ Department of Health Economics, School of Management and Medical Informatics, Tabriz University of Medical Sciences, Tabriz, Iran.; ^7^ Institute for Global Heath, University College London, London, UK.

## Dear Editor,

Backing by strong political support of the government, Ministry of Health and Medical Education (MoHME) launched a major and ambitious reform in Iran on May 5, 2014 entitled “Health Transformation Plan (HTP)” to address the main challenges in the healthcare system including high levels of out-of-pocket (OOP) payments, irrational medical tariffs, and low quality healthcare services.^1,2^ HTP aimed to address these issues through a number of strategies. These included increasing government spending on health, extending health insurance coverage, increasing medical tariffs, creating new hospitals in deprived regions and increasing hospital beds and recruiting more physicians and health workers, such as nurses and midwifes, in remote areas as well as repairing and renovating public hospitals and health facilities.^3,4^ HTP intended to cover four domains including medical services, public health and primary healthcare, medical education, and pharmaceutical products.

Aiming to implement HTP, the government injected more than 85 000 billion Iranian Rials (US$3196 million or INT$ 10 316 million^ [1] ^) additional funds into the healthcare system. The additional funds were generated by allocating 10% of targeted subsidies and 1% of value-added tax to the healthcare system and by increasing (though not considerable) the share of healthcare budget from governmental budget. In November 2014, as the third step of HTP, medical tariffs increased on average by 120% in order to tackle informal payments and making medical tariffs more realistic. As a result, the total health expenditure rose by 39% and its share as % of gross domestic product rose from 6.1% in 2013 to 8.13% in 2016 (Table). As shown in the Table, the shares of all financing sources have increased. For instance, OOP net amount has grown from 9.9% in 2013 to 18% in 2015 and has decreased to 2.2% in 2016. In contrast increase in the share of governmental health expenditures as percent of total governmental budget resulted in substantial reduction of OOP as % of total health expenditure, which peaked in the first year of HTP implementation with 9% reduction and with a slight decline afterward (Figure). Although the proportion of OOP has decreased, available evidence shows mixed results regarding the reduction in the incidence of catastrophic health expenditure.^5-8^ However, more rigorous research needed to explore the impact of HTP in this area.

**Table:** The Changes in Healthcare Expenditure During 2013-2016

	**2013**	**2014**	**2015**	**2016**
THE (in billion IRR)	621 623	864 604	1 036 846	1 146 138
THE % of GDP	6.1	7.5	8.7	8.13
THE annual growth (%)	-	39	20	10
Annual growth (%)				
Government^a^	58.8	85.8	9	12.4
SHI	41.7	69.9	39.2	20.9
OOP	9.9	14.7	18	2.2
Others	51.3	13.4	10.5	9.2
GHE % of government budget^b^	11.4	16.2	21	19

Abbreviations: IRR, Iranian Rials; THE, total health expenditure; GDP, gross domestic product; SHI, social health insurance; OOP, out-of-pocket; GHE, governmental health expenditure. ^a^ Government including Local, state and central Government based on National Health Accounts classification. ^b^ It is not equal to general government health expenditure (GGHE) % of general government expenditure (GGE). Source: National Health Accounts, MoHME.

**Figure:**
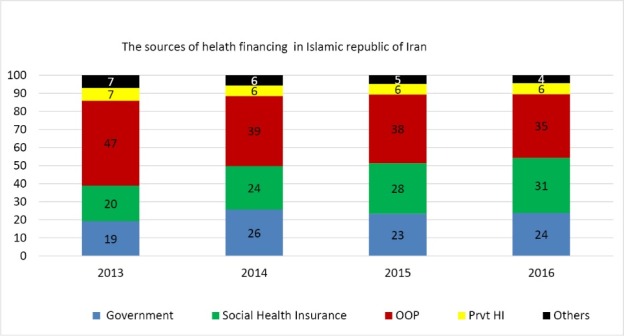
Changes in the Share of Different Sources of Total Health Expenditure as a Result of HTP. Abbreviations: HTP, Health Transformation Plan; OOP, out-of-pocket; Prvt HI: private health insurances. Source: National Health Accounts, MoHME.

By allocating 5500 billion Iranian Rials (2014 US$207 million and INT$ 668 million) to the Iran Health Insurance Organization (IHIO) by the government, free health insurance coverage was provided for uninsured individuals, who were mainly self-employed individuals living in urban areas. In 2014 nearly 11 million people were covered by IHIO and overall population coverage raised from 83% to 92%. The increase in insurance coverage, expanding the health insurance benefit package, and reduction in OOP, enhanced access to and utilization of the healthcare services, and in turn, led to satisfaction among the public.^8,9^ For instance, in 2015 and for patients covered by IHIO alone, utilization of inpatient services and average hospitalization costs increased by 6% and by more than 73% respectively.^10^

The rapid increase in the provision and utilization of healthcare services might be driven partly by unmet needs before the implementation of the HTP and partly because of consumer moral hazard, due to reduction in co-insurance rates in public hospitals, and providers’ induced demand, caused by increasing medial tariffs and open-ended fee-for-service payments.

Rising expenditures put high financial pressure on the government and public health insurance funds, including IHIO and social security organization, which led to long (up to one year) delays in reimbursing the hospitals and medical centers across the country. This situation put the government in a horrendous bind and made the MoHME to appeal to severe spending-cuts policies. For instance, MoHME forced medical universities to cut down their spending at least by 20% in 2018 compared to 2017.^10^

Alongside or even before the implementation of HTP, some other reforms to improve efficiency of healthcare system were required. These reforms included establishing evidence-informed demand and supply-side policies such as extending the referral system and family physician programme to urban areas and population segments other than rural residents, moving forwards to prospective provider payment mechanisms (such as capitation, diagnosis related groups, and pay for performance), applying clinical guidelines, as well as revising the benefit packages by including the most cost-effective interventions. Moreover, organizational reforms in public hospitals aim to balance between revenues and expenses is a prerequisite^11-13^ and HTP should try to affect structure and process of healthcare provision as well as culture and values of healthcare organizations to avoid over use and provider’s induced demand. HTP could have been used as a golden opportunity to implement the structural reforms, some mentioned above, which has been emphasized in the national development plans over the last decades.

The speed of policy reform process should also be taken into account. The new government began officially on August 3, 2013 and in less than one year, on May 5, 2014, the HTP as a major reform began, though with more focus on financing dimension of the healthcare system. It would have been better for the MoHME to take a more conservative approach and implement the HTP gradually or in some provinces as a pilot program first, explore potential impacts and identify its implementation pitfalls, and then scale it up at the national level after making necessary amendments in the plan. In launching a large-scale health reform such as HTP, if necessary considerations for long-term financial sustainability are not taken into account, the short-time achievements would not last long, and in long run, it can have negative impacts on the performance of healthcare system as a whole. However, with all its pitfalls and limitations, HTP is a window of opportunity to pave the way towards universal health coverage and the policy-makers should try to strengthen the plan, using the strategies mentioned above, and do not let this opportunity pass.

## Ethical issues

Not applicable.

## Competing interests

Authors declare that they have no competing interests.

## Authors’ contributions

LD and MB contributed to the conception of the work. LD drafted the letter. MB, BN, and HHB provided critical comments on the draft. All authors approved the final version.

## Authors’ affiliations

^1^Tabriz Health Services Management Research Center, Iranian Center of Excellence in Health Management, Tabriz University of Medical Sciences, Tabriz, Iran. ^2^Social Determinants of Health Research Center, Health Management and Safety Promotion Research Institute, Tabriz University of Medical Sciences, Tabriz, Iran. ^3^Department of Health Services Management, School of Management and Medical Informatics, Tabriz University of Medical Sciences, Tabriz, Iran. ^4^Department of Public Health, Faculty of Health, Ilam University of Medical Sciences, Ilam, Iran. ^5^Tabriz Health Services Management Research Center, Tabriz University of Medical Sciences, Tabriz, Iran. ^6^Department of Health Economics, School of Management and Medical Informatics, Tabriz University of Medical Sciences, Tabriz, Iran. ^7^Institute for Global Heath, University College London, London, UK.

## Endnote


[1] One dollar in US and International exchange rate (purchasing power parity) was equal to 26 594 and 8239 Iranian Rial in 2014, respectively (Source: International Monitory Fund, 2016).

